# IgG4-Related Esophageal Disease Presenting as Esophagitis Dissecans Superficialis With Chronic Strictures

**DOI:** 10.14740/jocmr1845w

**Published:** 2014-05-22

**Authors:** Myriam Dumas-Campagna, Simon Bouchard, Genevieve Soucy, Mickael Bouin

**Affiliations:** a Gastroenterology Unit, Hopital Saint-Luc du Centre Hospitalier de l’Universite de Montreal,1058 Saint-Denis, Montreal H2X 3J4, Canada; b Pathology Department, Hopital Saint-Luc du Centre Hospitalier de l’Universite de Montreal,1058 Saint-Denis, Montreal H2X 3J4, Canada

**Keywords:** IgG4-related disease, Esophagitis dissecans, Sclerosing esophagitis, Autoimmune esophagitis

## Abstract

IgG4-related disease is a recently recognized autoimmune systemic disorder that has been described in various organs. The disease is characterized histologically by a dense lymphoplasmocytic infiltrate of IgG4-positive cells, storiform fibrosis and can be associated with tumefactive lesions. IgG4-related disease involving the upper gastrointestinal tract is rare and only two previous case reports have reported IgG4-related esophageal disease. We report the case of a 63-year-old female patient with a long-standing history of severe dysphagia and odynophagia with an initial diagnosis of reflux esophagitis. Symptoms persisted despite anti-acid therapy and control esophagogastroduodenoscopy (EGD) revealed endoscopic images consistent with esophagitis dissecans superficialis (sloughing esophagitis). An underlying autoimmune process was suspected and immunosuppressant agents were tried to control her disease. The patient eventually developed disabling dysphagia secondary to multiple chronic esophageal strictures. A diagnosis of IgG4-related disease was eventually made after reviewing esophageal biopsies and performing an immunohistochemical study with an anti-IgG4 antibody. Treatment attempts with corticosteroids and rituximab was not associated with a significant improvement of the symptoms of dysphagia and odynophagia, possibly because of the chronic nature of the disease associated with a high fibrotic component. Our case report describes this unique case of IgG4-related esophageal disease presenting as chronic esophagitis dissecans with strictures. We also briefly review the main histopathological features and treatment options in IgG4-related disease.

## Introduction

IgG4-related disease has recently been recognized as an autoimmune systemic disorder. The first reports of the disease came from Japan where it was thought that autoimmune pancreatitis associated with high serum concentration of IgG4 and extra-pancreatic manifestations might be part of a more systemic autoimmune disorder [1]. IgG4-related disease is diagnosed histologically as a dense lymphoplasmocytic infiltrate with IgG4-positive cells and fibrosis organized in a storiform pattern [2]. Most patients have a high concentration of IgG4 in the serum. The disease can also be associated with mass-like lesions that may be mistaken for cancerous lesions [3]. Many organs can be involved by the disease such as the pancreas, biliary tract, salivary glands, lymph nodes, thyroid, kidneys, lung, skin, prostate and aorta [4]. Involvement of the upper gastrointestinal (GI) tract is rare and there has only been two case reports describing IgG4-related esophageal disease [5, 6]. We report a case of IgG4-related esophageal disease presenting as chronic esophagitis dissecans with strictures.

## Case Report

A 63-year-old Caucasian woman was evaluated for a 10-year history of progressive odynophagia and dysphagia. Her medical history was positive for primary biliary cirrhosis, Sjogren disease, Raynaud disease, asthma, transient cerebral ischemia and osteoporosis.

This patient was initially evaluated in gastroenterology in 2001 for symptoms of heartburn and dyspepsia. An esophagogastroduodenoscopy (EGD) done at that moment revealed a distal oesophagitis (Los Angeles grade A) with superficial erosions. The presumed diagnosis was gastroesophageal reflux disease (GERD) and she was treated with esomeprazole, a proton pump inhibitor, at a standard daily dose.

After an initial improvement of 2 years, she experienced recurrent episodes of odynophagia and dysphagia localized behind the lower sternum. In 2004, she had a second EGD that was normal. An esophageal manometry and a gastric emptying test were also normal. However, an ambulatory intraesophageal pH monitoring under esomeprazole revealed significant acid reflux. The patient was then treated with a twice-daily dose of pantoprazole. A coloscopy done for symptoms of constipation was also normal.

Despite this standard treatment, odynophagia persisted and was associated with weight loss. In spring 2005, two other EGD at 2-month interval showed a circumferential severe ulcerative esophagitis on the lower two-thirds of the esophagus. Esophageal biopsies excluded any fungal or viral infection and demonstrated only non-specific inflammation without eosinophilia. Bullus pemphigus was excluded by the absence of separation of the suprabasal level of epithelium on biopsy specimen and the absence of clinical history of skin involvement. Absence of bowel symptoms, anormal abdominal CT scan and a second negative colonoscopy made inflammatory bowel disease unlikely. Serologies and capillaroscopy were negative for connective tissue disorders such as scleroderma. A thoracic and abdominal CT scan did not reveal sign of neoplasia except for four pulmonary nodules of less than 5 mm. Two of them were adjacent in the right middle lobe and the others were in different segments of the right lower lobe. These nodules were considered benign because they did not grow after several months of follow-up and a bronchoscopy was normal.

In winter of 2006, she was hospitalized for severe odynophagia and progressive dysphagia. The EGD revealed ulcerations with a friable and sloughing mucosa on the distal two-thirds of the esophagus, an image consistent with esophagitis dissecans superficialis. The esophageal biopsies showed non-specific active ulcerative esophagitis with chronic inflammation. The immunohistologic study demonstrated CD3+ T lymphocytes, CD20+ B lymphocytes and plasmocytes. Again, investigations were negative for an infectious cause but a short treatment with acyclovir was attempted, without significant improvement of her symptoms. A possible diagnosis of autoimmune esophagitis was evoked and treatment with oral prednisone induced remission of her symptoms. Unfortunately, symptoms reappeared during attempts of prednisone withdrawal. In December 2006, oral mercaptopurine was prescribed for maintenance therapy and as a glucocorticoid-sparing agent. Because of severe nausea with this medication, she was switched to oral mycophenolate mofetil. During the following months, she continued experiencing disabling nausea, weight loss and was unable to taper prednisone. In fall of 2007, mycophenolate mofetil was stopped and oral cyclosporine was introduced. This led to stabilization of her weight with slight improvement of dysphagia and odynophagia. Prednisone was also tapered but could not be completely stopped.

In spring of 2008, she experienced an episode of severe acute ulcerative esophagitis. An EGD revealed diffuse ulcerations, sloughing of the mucosa and a long esophageal narrowing. Biopsies showed inflammatory fibrinous exudate with granulation tissue. Because of intolerance to an oral diet, a percutaneous gastrostomy was installed for enteral feeding. Cyclosporin was stopped and she was treated with infliximab and prednisone. With infliximab, her symptoms improved and she was able to tolerate an oral diet. In summer of 2010, she was switched to adalimumab because of an infusion reaction with infliximab.

**Figure 1:**
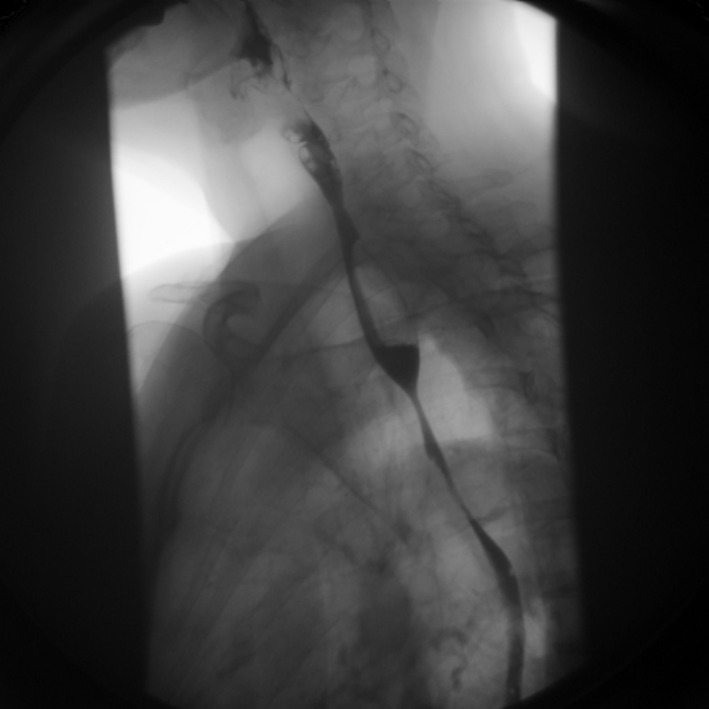
Barium swallow showing a short esophageal stenosis at the C5-C6 level and a long esophageal stenosis in the mid esophagus.

**Figure 2:**
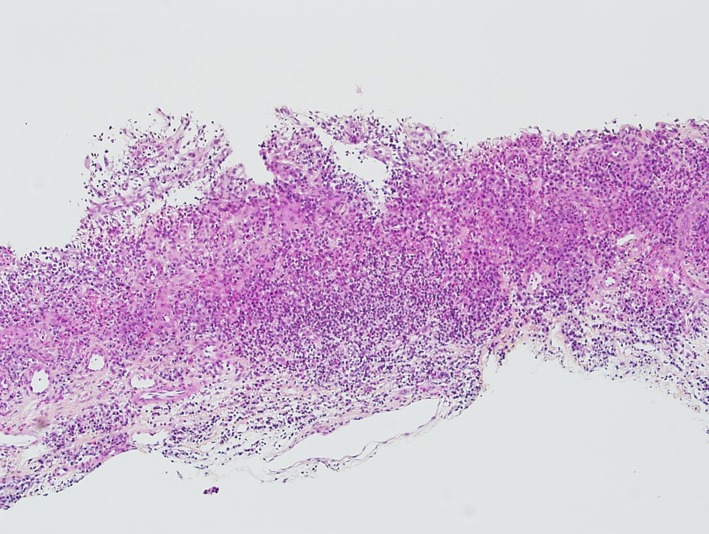
Histologic image of an esophageal biopsy (× 10) revealing a dense lymphoplasmocytic infiltrate. Hematoxylin phloxine saffron stain.

**Figure 3:**
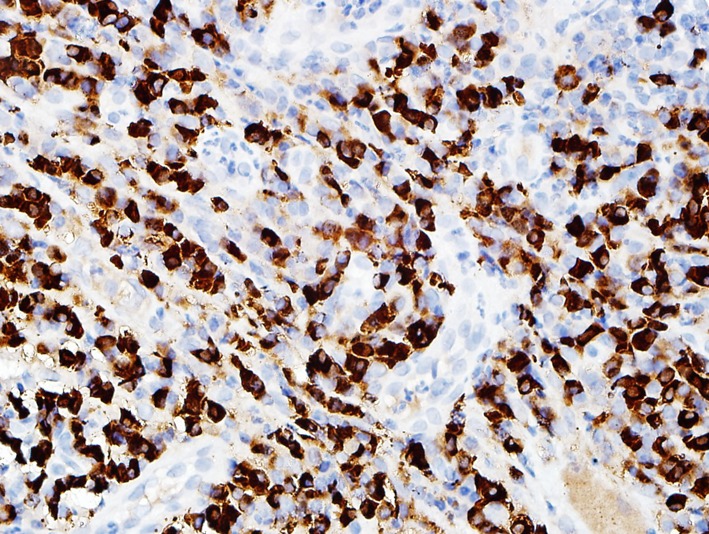
Histologic image (× 40) of the esophagus with many IgG4-positive plasma cells.

In spring of 2011, the patient had severe dysphagia and was unable to swallow saliva. An EGD could not be performed due to tight esophageal stenosis. A barium swallow confirmed the presence of three stenosis at different levels of the esophagus. The first one was 1 cm long at the cervical level (C5-C6), the second one was 7 cm long at the thoracic level (Fig. 1) and the last one was 3 cm long in the distal esophagus. Her 2006 esophageal biopsies were reviewed with a pathologist and an immunohistochemical study with an anti-IgG4 antibody was done (Fig. 2, 3). The presence of 30 IgG4-positive plasma cells per high-power field was consistent with a diagnosis of IgG4-related esophageal disease. Adalimumab was stopped at that moment because of disease progression under treatment. Rituximab, a monoclonal antibody against the CD20 protein, was then attempted with two intravenous doses of 1 g at 2-week interval. Unfortunately, there was no significant clinical improvement.

In the past months, the patient had persistent severe dysphagia and odynophagia. She still received enteral nutrition through a gastrostomy. During acute flares of symptoms, she was treated initially with prednisone at a dose of 40 mg per day and tapered thereafter. There seemed to be a small response to those short courses of steroids.

In the morning of March 3, 2013, she was found with altered consciousness and was brought to the regional hospital. In the emergency room, she was evaluated with a Glascow of three of unclear reason. Decision was made to follow her wishes not to be reanimated and she passed away a few hours later on confort care. Autopsy was accepted by the family and could not identify the reason of her death. The autopsy was performed in another hospital and confirmed the presence of esophageal stenosis and primitive biliary cirrhosis at a fibrosis stage of three. It also demonstrated important heart atherosclerosis but without acute infarctand there was no acute intracranial event. Few representative esophageal sections were submitted for microscopic evaluation in our hospital. Some sections showed thickening of the esophageal wall with complete absence of squamous mucosa associated with lamina propria sclerosis and muscularis mucosae hypertrophy. Few inflammatory cells, mainly lymphocytes and plasma cells, were found in the lamina propria, associated with sclerosis, contrasting with the 2006 biopsies where inflammatory cells were prominent. One esophageal section was completely normal without any thicknening of the wall or any changes in the mucosa. IgG4 immunostains were performed on all esophageal sections. Compared to the 2006 esophageal biopsy, very few IgG4-positive cells were noted on the auptosy sections.

## Discussion

This is a 63-year-old woman with IgG4-related esophageal disease presenting as esophagitis dissecans superficialis with chronic strictures.

In 2011, Lee et al [5] described the first case of IgG4-related sclerosing esophagitis. Their patient had progressive dysphagia and weight loss and the diagnosis was made by histologic study of the esophagectomy specimen.

Corticosteroids are used as initial therapy for IgG4-related disease. Initial dosage of 0.6 mg/kg has been suggested in a 2012 Japanese consensus for treatment of symptomatic autoimmune pancreatitis, which included patients with symptomatic extrapancreatic manifestations [7]. After 2 - 4 weeks, the dose is gradually tapered every 1 - 2 weeks until a maintenance dose of 2.5 - 5 mg of prednisolone is achieved. Corticosteroids can be stopped completely after a few months if the patient does not have residual active disease, but there is a high rate of relapse.

Immunomodulators such as azathioprine, methotrexate and mycophenolate mofetil can be used as maintenance therapy and as a steroid-sparing strategy in patients refractory to or dependant of corticosteroid therapy. Rituximab, a monoclonal antibody directed at CD20 antigen on B-lymphocytes, has recently been used in a few patients with disease refractory to standard treatment [8, 9].

In our patient, the use of rituximab was not associated with a significant improvement of the symptoms of dysphagia and odynophagia. One possible explanation is that because of the long duration of the symptoms, the esophageal strictures are primarily fibrotic rather than inflammatory. It has been suggested that IgG4-related disease with a high fibrotic component may respond less to treatment with corticosteroids and immunomodulators such as rituximab [8].

The diagnosis of IgG4-related esophageal disease was made 10 years after the initial evaluation in gastroenterology. Initial complaints and endoscopic findings were compatible with GERD. However, there was incomplete clinical response to standard therapy with proton pump inhibitors.

During the following years, the patient developed recurrent episodes of severe esophageal symptoms with endoscopic findings compatible with esophagitis dissecans superficialis and multiple ulcerations.

Many diseases affecting the esophagus can cause esophagitis dissecans: autoimmune bullous dermatosis (pemphigus vulgaris, bullous pemphigoid, Stevens-Johnson disease) [10], celiac disease [11] and post-sclerotherapy for esophageal varices [12]. Cases have also been reported with the use of biphosphonates [13, 14], NSAIDs and potassium chloride [15]. In most patients, however, esophagitis dissecans is idiopathic and this seemed to be the case in our patient. Although a biopsy of the mouth mucosa in July 2011 revealed histologic findings compatible with lichen planus, these findings were never seen on the patient’s esophageal biopsies.

The patient’s medical history and the repetitive endoscopic findings of esophageal mucosal sloughing, inflammation and persistent ulcerations led us to believe that an underlying autoimmune process was responsible for her esophageal symptoms. As we previously described, different immunosuppressant agents were tried to control her disease.

In 1997, Ponsot et al [16] described a series of five patients with chronic dysphagia and detachment of the esophageal mucosa either spontaneously or after passage of the scope. He proposes the term chronic esophagitis dissecans for these patients. One of the histologic characteristics of this new entity was the relative absence of inflammation. Although the clinical and endoscopic description could apply to our patient, there was recurrent inflammation at biopsies indicating an underlying inflammatory disorder.

In 2011, IgG4-related disease was suspected due to the patient’s history of autoimmune disease and due to the increasing literature describing this recently recognized condition that can involve different organs. It is only after reviewing the esophageal biopsies that were initially done in 2006 and using a specific immunohistological study for IgG4 that we diagnosed IgG4-related disease involving the esophagus.

New sets of biopsies after treatment could not be done because of the severe esophageal strictures that made it impossible to intubate the esophagus. The low count of IgG4-positive cells on esophageal tissue at autopsy may be explained by the chronic course of the disease and the predominance of fibrosis.

To our knowledge, this is the second case of IgG4-related esophageal disease presenting as esophagitis dissecans with strictures. Since IgG4-related disease is a recently described disorder, it is possible that IgG4-related esophageal disease is an underdiagnosed entity.
